# Apolipoprotein E (APOE) regulates the transport of monosialotetrahexosylganglioside (GM1)

**DOI:** 10.1016/j.jbc.2025.110778

**Published:** 2025-09-27

**Authors:** Dong Yan Zhang, Jian Wang, Gangtong Huang, Martin Dokholyan, Smaranda Willcox, Jack Griffith, Feng Ding, Nikolay V. Dokholyan

**Affiliations:** 1 Department of Neurology, University of Virginia, School of Medicine, Charlottesville, Virginia, USA; 2 Department of Physics and Astronomy, Clemson University, Clemson, South Carolina, USA; 3 Department of Neuroscience and Experimental Therapeutics, Penn State College of Medicine, Hershey, Pennsylvania, USA; 4 Lineberger Comprehensive Cancer Center and Departments of Microbiology and Immunology, and Biochemistry and Biophysics, The University of North Carolina at Chapel Hill, Chapel Hill, North Carolina, USA; 5 Department of Biomedical Engineering, University of Virginia, School of Medicine, Charlottesville, Virginia, USA; 6 Department of Neuroscience, University of Virginia, School of Medicine, Charlottesville, Virginia, USA

**Keywords:** APOE, GM1 ganglioside, lipid transport, Alzheimer's disease, amyloid beta aggregation

## Abstract

Apolipoprotein E (APOE) is a key lipid transporter involved in the trafficking and clearance. The ε4 allele of APOE (APOE4) is the strongest genetic risk factor for late-onset Alzheimer’s disease (AD), and certain lipids are closely linked to AD pathology. APOE may contribute to AD pathogenesis through its lipid transport function. Although cholesterol is a well-established cargo of APOE and has been associated with AD, its role as a mechanistic link between APOE and AD has not been demonstrated. Here, we demonstrate that monosialotetrahexosylganglioside (GM1), a membrane lipid implicated in AD, is a preferential binder of APOE. We have previously shown that GM1 promotes amyloid beta (Aβ) oligomer aggregation, which is a critical step in AD pathology. Here, we show that APOE binds GM1 with higher affinity than cholesterol and facilitates greater cellular uptake of GM1-containing lipid structures in a cell-type-dependent manner. Furthermore, GM1 alters the secondary structure of APOE and enhances its interaction with the low-density lipoprotein receptor, thereby promoting the internalization of lipid assemblies. Using confocal imaging and discrete molecular dynamics simulations, we show that membranes containing 20% GM1 form stable stripe-like clusters, consistent with the formation of GM1-enriched lipid rafts that may serve as physiological platforms for APOE:GM1 interactions. These results reveal a reciprocal relationship in which APOE regulates GM1 transport, while GM1 modifies APOE function and localization. The competition between GM1 and cholesterol for APOE binding may contribute to cholesterol dysregulation in APOE4 carriers. Our results uncover a novel mechanistic link between APOE and AD pathogenesis through GM1-mediated promotion of Aβ aggregation.

APOE (apolipoprotein E; 299 amino acids, ∼34 kDa) is a key lipid-transport protein, primarily synthesized and secreted by glial cells, especially astrocytes (1). It plays essential roles in lipid metabolism, neuronal repair, and synaptic plasticity. Following synthesis, lipid-free APOE undergoes lipidation (2, 3) to form lipoproteins, such as high-density lipoprotein particles (4, 5), delivering cholesterol and phospholipids to neurons *via* interactions with cell surface receptors (*e.g.*, low-density lipoprotein receptor (LDLR) and LRP1). Through these processes, APOE supports synaptic function and structural integrity. Among the three human isoforms (APOE2, APOE3, and APOE4), APOE4 is a major genetic risk factor for late-onset Alzheimer’s disease (AD) (6). Although the connection between APOE4 and AD has been extensively studied, the underlying mechanisms remain unclear.

One hypothesis is that APOE contributes to AD pathogenesis through its lipid transport function, as certain lipids (7, 8, 9, 10) are closely linked to AD pathology. For example, cholesterol is a well-established cargo of APOE and has been associated with AD. Individuals with elevated levels of plasma cholesterol have an increased susceptibility to AD (11, 12). Extracellular cholesterol has been found to strengthen amyloid beta (Aβ) fibrils and oligomers (13). The aggregation of Aβ is a hallmark of AD, and Aβ pathology is considered a central feature of AD (14, 15). Free cholesterol interacts with specific residues in Aβ peptides (16, 17). However, the role of cholesterol as a mechanistic link between APOE and AD has not been conclusively demonstrated.

Here, we show that monosialotetrahexosylganglioside (GM1), a membrane lipid implicated in AD, is a preferential binder of APOE. GM1, a ganglioside highly abundant in the nervous system (8, 18), is involved in Aβ aggregation and neuronal protection (19). We have previously shown that GM1 promotes the aggregation of Aβ oligomers. GM1 is enriched in specialized microdomains (20) within cell membranes called lipid rafts. The ganglioside content in the frontal cortex of early stage AD patients is similar to age-matched controls, but the proportion of gangliosides localized in lipid rafts, particularly GM1, doubles (18, 21). Similarly, platelets from patients with AD exhibit significantly higher GM1 content in lipid rafts than those from controls, further implicating GM1 in AD pathology (18). Other studies (9, 22, 23, 24) have consistently highlighted the critical role of GM1 in Aβ pathologies, linking this ganglioside to the progression of AD (22, 23, 25). Indeed, early findings of GM1 led to numerous clinical trials in AD among other conditions (26, 27, 28, 29, 30, 31).

The involvement of GM1 and cholesterol in AD pathogenesis underscores the multifaceted role of lipids in AD progression. Although APOE is well-established as a key transporter of cholesterol, its potential role in GM1 regulation remains largely unexplored. Prior studies have indicated an age-related increase in GM1 levels within detergent-resistant membranes in the brains of APOE4 knock-in mice (19, 32). However, direct connections between APOE and GM1 remain poorly understood. Here, we propose that APOE facilitates the transport of GM1, influencing Aβ pathology. We employ a multifaceted approach, integrating *in vitro*, *in silico*, and cellular studies to examine the interaction between GM1 and APOE. Particularly, we combine *in vitro* binding assays, cellular uptake experiments, circular dichroism spectroscopy, and discrete molecular dynamics (DMD) simulations. We find that APOE interacts with GM1-containing lipid structures in ways that influence lipid transport to the cell membrane and modulate both APOE structure and its receptor binding. We further determine how GM1 clustering on the membrane affects lipid raft stability and contributes to lipid uptake dynamics. Our findings suggest a bidirectional interplay between APOE and GM1: APOE regulates the distribution of GM1 on membranes, while GM1, in turn, modulates APOE’s structural conformation and cellular transport behavior. These insights point to a previously unrecognized lipid-mediated mechanism by which APOE may influence AD pathogenesis.

## Results

### GM1-containing lipid structures exhibit higher binding affinity relative to cholesterol-containing structures

To elucidate the potential regulatory role of APOE in GM1 transport, we measured the binding affinity between APOE and GM1, comparing it to the affinity of APOE with cholesterol and other lipids commonly found in membrane lipid structures. Lipid structures were prepared using cholesterol, GM1, sphingomyelin (SM), and l-α-phosphatidylcholine (PC). Both APOE3 and APOE4 were labeled with RED-NHS 2nd Generation, a dye containing a reactive NHS-ester group that forms a covalent bond with primary amines (lysine residues). APOE contains 13 lysine residues, including two within the lipid-binding region aa 244 to 272 (33). Random labeling was used to avoid any potential bias caused by labeling at a specific lysine residue, as it ensures a population of APOE molecules labeled at various sites, mitigating any site-specific alterations to the protein's properties.

**Figure 1:**
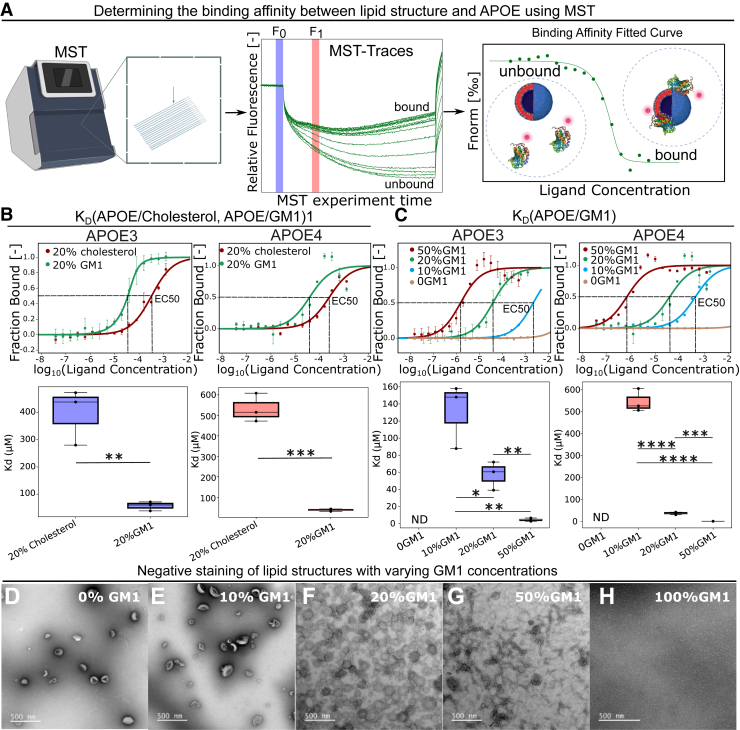
**Determination of the interaction affinity between GM1 and APOE.***A*, schematic illustration of determining the binding affinity between lipid structure and APOE using MST. *B*, the binding affinity (K_D_) between APOE3 or APOE4 and lipid structures containing GM1 or cholesterol (n = 3). *C*, the binding affinity between APOE3 or APOE4 and lipid structures with different GM1 concentrations. (n = 3). *D*-*H*, negative staining images of lipid structures with varying GM1 concentrations. *p* value: ns (0.05 < *p* ≤ 1), ∗ (0.01 < *p* ≤ 0.05, ∗∗ (0.001 < *p* ≤ 0.01, ∗∗∗ (0.0001 < *p* ≤ 0.001, ∗∗∗∗ (*p* ≤ 0.0001). (*A*) is created with BioRender.com. APOE, apolipoprotein E; MST, microscale thermophoresis.

We employed microscale thermophoresis (MST) to determine the binding affinity between various lipid structures and both APOE3 and APOE4. Given the intricate nature of lipid bilayers and the possibility of certain lipids being encapsulated within liposomes, the precise effective concentration of lipids available for binding to APOE remains ambiguous. As a result, the dissociation constant (K_D_) values reported are relative values. Our findings demonstrated that GM1 exhibited a stronger binding affinity to both APOE3 and APOE4 compared with cholesterol (Figs. 1, *A*, *B* and S1). Considering APOE's role in cholesterol transport and GM1's enhanced affinity to APOE over cholesterol, the results suggest a potential involvement of APOE in GM1 transport.

### The binding affinity between APOE and lipid structures with different GM1 contents

To assess the impact of different GM1 concentrations on the properties of lipid structures, we first characterized the morphology and size distribution of these structures through negative staining and dynamic light scattering techniques, as illustrated in Figures 1, *D*–*G*, S2, and S3. With 0% or 10% GM1, the lipid structures formed disc-shaped assemblies. At 20% GM1, they formed liposomes. When GM1 content increased to 50%, the lipid structures transitioned to elongated micelles, and at 100% GM1, they formed spherical micelles. To focus on physiologically relevant conditions, we limited our subsequent experiments to GM1 concentrations that more closely mimic biological systems, excluding the 100% GM1 condition. We also characterized the zeta potential of the lipid structures (Fig. S4). The results indicate that GM1 concentration significantly influences surface charge, though the relationship between GM1 content and zeta potential is nonlinear. The effects of GM1 on surface charge depend on various factors, including lipid composition and the formation of distinct lipid domains. Notably, at 20% GM1, a stronger negative charge is observed, suggesting that GM1 may promote specific lipid organization or clustering, resulting in a more defined and stable surface charge at this concentration.

To elucidate the influence of GM1 concentrations in membranes on the binding affinity of GM1 with APOE, we assessed the binding affinities between APOE3 and APOE4 and the lipid structures across a range of GM1 concentrations (0%, 10%, 20%, and 50%) (Figs. 1*C* and S1). Our analysis indicates that the absence of GM1 in the lipid structures results in undetected binding. The incorporation of elevated concentrations of GM1 within lipid structures has been shown to enhance the binding affinity for both APOE3 and APOE4. This finding suggests that the binding affinity between the GM1 membrane and APOE is significantly influenced by the concentration of GM1.

In the context of binding affinity assays conducted using MST, lipid structures serve as ligands. Nevertheless, challenges associated with lipid aggregation, fusion, and the formation of multilamellar vesicles at elevated lipid concentrations hinder our ability to further augment the ligand concentration, thereby preventing the attainment of a plateau in certain binding curves. Despite this, K_D_ and half-maximal effective concentration (EC_50_) are still applicable for comparing binding affinities across different binding curves. Notably, we observe a reduction in the binding signal for lipid structures containing 50% GM1, particularly in the case of APOE4 binding—an effect that is still detectable even at 20% GM1. This phenomenon likely stems from two factors: (1) membrane destabilization caused by high GM1 concentrations (50%), which prevented us from increasing lipid concentrations further; and (2) the inherent conformational rigidity of APOE4, which impedes its ability to adapt to dynamic lipid membrane surfaces as lipid structure concentrations rise, thereby reducing the availability of accessible binding sites.

### APOE influences the cellular uptake of GM1 lipid structures

**Figure 2:**
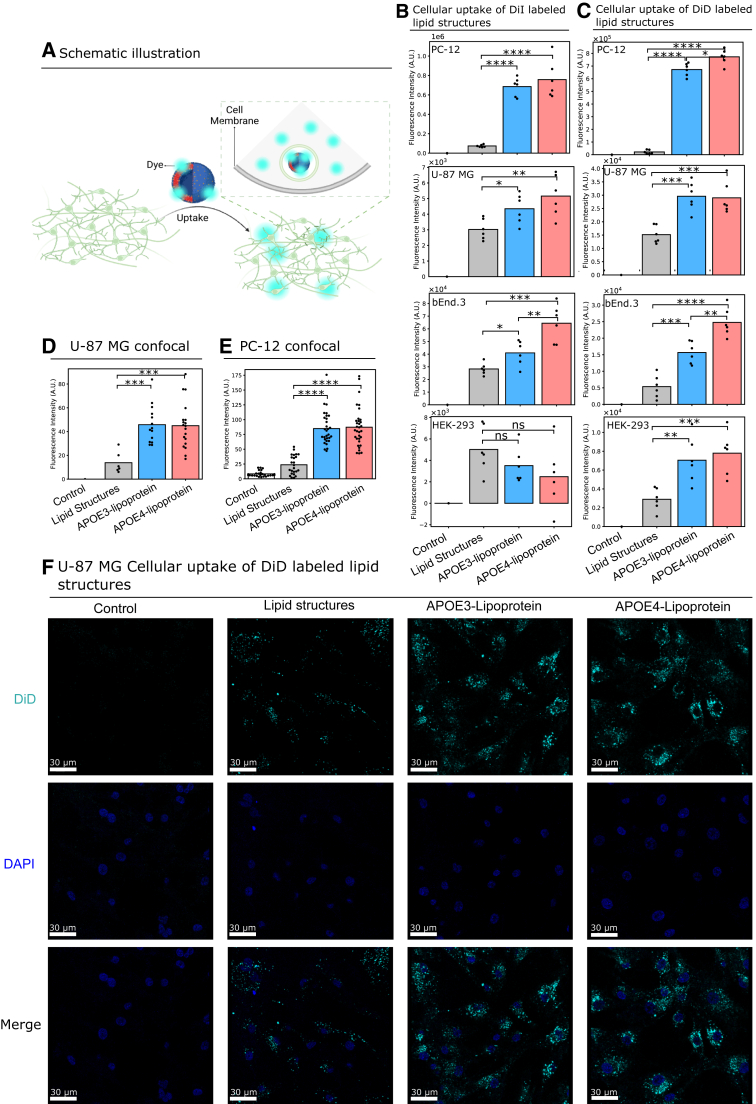
**Determination of the cellular uptake of lipid structures.***A*, schematic illustration of the method used to determine cellular uptake by labeling lipids with DiI or DiD. *B*, cellular uptake of DiI labeled lipid structures on differentiated PC-12 cells, U-87 MG, bEnd.3, and HEK-293 cells. (n ≥ 6) (*C*) cellular uptake of DiD-labeled lipid structures on differentiated PC-12 cells, U-87 MG, bEnd.3, and HEK-293 cells. (n ≥ 6) (*D*) quantification of the confocal microscopy images of U-87 MG cellular uptake of DiD-labeled lipid structures using ImageJ. *E*, quantification of the confocal microscopy images of PC-12 cellular uptake of DiD-labeled lipid structures using ImageJ. *F*, representative images showing cellular uptake of lipid structures labeled with DiD, as determined by confocal microscopy. (n ≥ 6). *p* value: ns (0.05 < *p* ≤ 1), ∗ (0.01 < *p* ≤ 0.05, ∗∗ (0.001 < *p* ≤ 0.01, ∗∗∗ (0.0001 < *p* ≤ 0.001, ∗∗∗∗ (*p* ≤ 0.0001). (*A*) is created with BioRender.com. Created in BioRender. Dokholyan, N. (2025) https://BioRender.com/g17z389. HEK, human embryonic kidney; PC, l-α-phosphatidylcholine.

To determine whether APOE transports GM1 to the cell membrane, we studied the influence of APOE on the cellular uptake of GM1 lipid structures. Lipid structures containing varying GM1 contents and labeled with DiI were incubated with APOE3 and APOE4 overnight at 37 °C, resulting in the APOE-enriched lipoproteins. Subsequently, these lipoproteins were incubated with differentiated PC-12 cells. After removing the excess lipoproteins from the culture medium, we assessed changes in the fluorescence intensity of DiI on the cells. We found a significant increase in cellular uptake when lipid structures contained 20% GM1 in the presence of both APOE3 and APOE4. This is attributed to several factors, including the morphology and stability of the lipid structures. Lipid structures containing 20% GM1 form well-defined liposomal shapes as shown in Figure 1, *D*–*G*, which are more favorable for cellular uptake. In addition, zeta potential results reveal that lipid structures containing 20% GM1 exhibit enhanced stability compared to those with other GM1 concentrations. Although higher GM1 levels generally increase the binding affinity between lipid structures and APOE, efficient cellular uptake also depends on the structural properties and overall stability of the lipid structures. To further validate the regulatory effect of APOE on GM1, we determined the cellular uptake of 20% GM1 lipid structures with human embryonic kidney (HEK)-293, U-87 MG, and bEnd.3 cells. U-87 MG and bEnd.3 cell lines are reported to express high levels of APOE receptors (34). DiI fluorescence intensity analysis demonstrated a significant increase in fluorescence intensity in the presence of APOE3 and APOE4 for U-87 MG and bEnd.3 cells, similar to the observations in differentiated PC-12 cells (Fig. 2, *A* and *B*). However, in HEK-293 cells, the fluorescence intensity exhibited no significant change.

We also employed DiD labeling to evaluate the cellular uptake of lipid structures. The results showed that, across all cell types, including differentiated PC-12 cells, U-87 MG, bEnd.3, and HEK-293 cells, the presence of APOE3 and APOE4 enhances the cellular uptake of GM1 lipid structures (Fig. 2*C*). In concert with the DiI-labeled outcomes, differentiated PC-12 cells exhibited the most substantial uptake of lipid structures, and U-87 MG and bEnd.3 cells displayed increased cellular uptake in the presence of APOE3 and APOE4. In contrast to DiI-labeled cellular uptake, HEK-293 cells exhibited significant cellular uptake of DiD-labeled lipid structures in the presence of APOE3 and APOE4. However, when compared to other cell types, HEK-293 cells demonstrated the least amount of GM1 lipid structure uptake. The discrepancy may be attributed to the lower level of cellular uptake in HEK-293 cells, as well as the lower sensitivity of DiI compared to DiD Furthermore, APOE4 exhibited significantly higher cellular uptake than APOE3 in differentiated PC-12 and bEnd.3 cells. In contrast to the control with lipid structures, the difference between APOE3 and APOE4 was relatively minor, and no significant distinction was noted when DiI was employed for labeling. These findings indicate that the capacity to discern the differential effects of APOE3 and APOE4 on cellular uptake may be influenced by various experimental factors, including the type and sensitivity of the dye utilized. To visually assess the cellular uptake of GM1 lipid structures, we labeled the lipid structures with DiD and used confocal microscopy to observe uptake by differentiated PC-12 and U-87 MG cells. Quantification of the images was performed using ImageJ. The results show that, for both differentiated PC-12 and U-87 MG cells, the presence of APOE3 and APOE4 enhances cellular uptake (Figs. 2, *D*–*F* and S5). Remarkably, APOE4 exhibits significantly higher cellular uptake than APOE3 in differentiated PC-12 cells. These findings are consistent with the previously DiD-labeled fluorescence results. The facilitation of cellular uptake by APOE implies its regulatory role in GM1 transport.

The results of our analysis indicate that the presence of APOE3 and APOE4 plays a crucial role in regulating the transport of GM1 to the cell membrane. The efficiency of this transport is contingent upon the specific cell type, and the effectiveness of APOE3 and APOE4 exhibits variability across different cell lines.

### Localization of GM1 following cellular uptake

**Figure 3:**
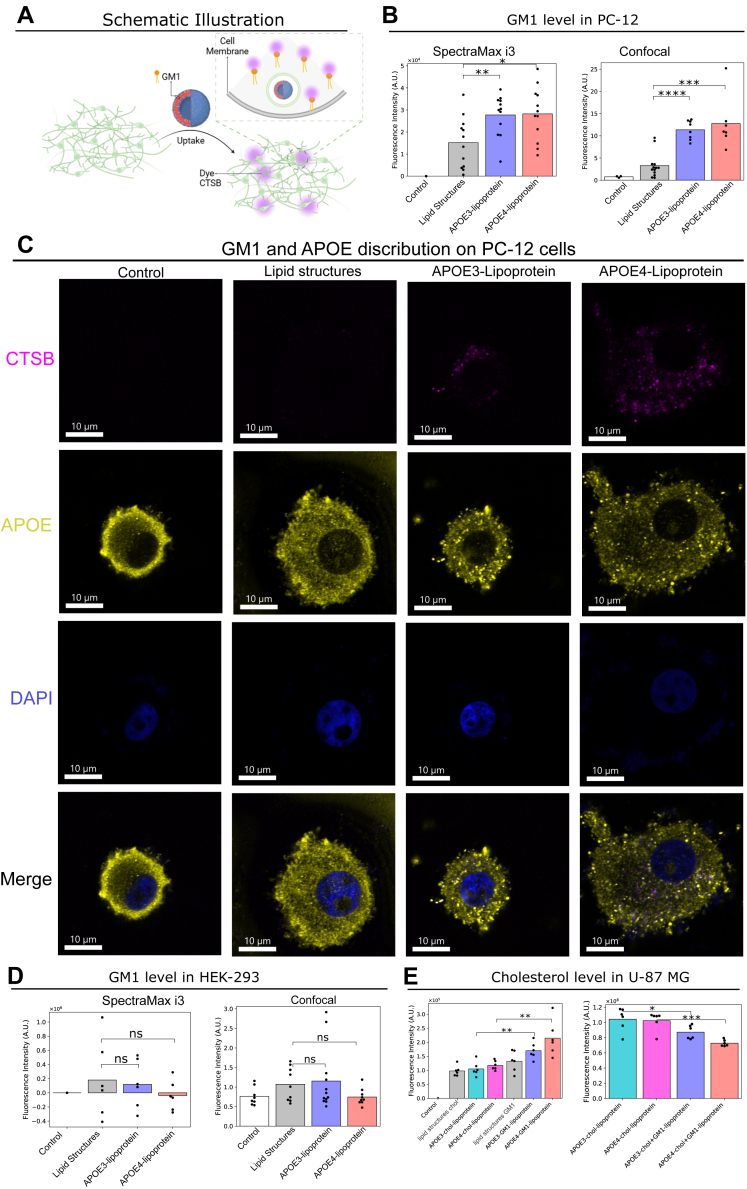
**Analysis of GM1 localization and levels following the cellular uptake of lipid structures.***A*, schematic illustration of the method used to determine GM1 following the cellular uptake. *B*, GM1 levels in differentiated PC-12 cells following cellular uptake, determined using the SpectraMax i3 or by quantifying confocal microscopy images. (n ≥ 3) (*C*) representative images showing GM1 and APOE localization in differentiated PC-12 cells following cellular uptake, as determined by confocal microscopy. (n ≥ 6) (*D*) GM1 levels in HEK-293 cells following cellular uptake, determined using the SpectraMax i3 or by quantifying confocal microscopy images. (n ≥ 6) (*E*) (1) cellular uptake of DiD-labeled cholesterol and GM1 lipid structures in U-87 MG cells, and (2) changes in cholesterol levels in U-87 MG cells after cellular uptake of APOE3 and APOE4-enriched cholesterol lipoproteins, as well as a mixture of APOE3 and APOE4-enriched cholesterol and GM1 lipoproteins. (n ≥ 6). *p* value: ns (0.05 < *p* ≤ 1), ∗ (0.01 < *p* ≤ 0.05, ∗∗ (0.001 < *p* ≤ 0.01, ∗∗∗ (0.0001 < *p* ≤ 0.001, ∗∗∗∗ (*p* ≤ 0.0001). (*A*) is created with BioRender.com. Created in BioRender. Dokholyan, N. (2025) https://BioRender.com/g17z389. APOE, apolipoprotein E; HEK, human embryonic kidney; PC, l-α-phosphatidylcholine.

To gain a quantitative understanding of the distribution and precise changes in GM1 following the cellular uptake of GM1 lipid structures, we utilize Cholera Toxin Subunit B, Alexa Fluor 555 Conjugate (CTSB 555) to label and determine the GM1 content on the cell membrane. The procedure involved the incubation of APOE-GM1 lipoproteins with differentiated PC-12 cells, with the removal of surplus APOE-GM1 lipoproteins from the culture medium. We find that the presence of both APOE3 and APOE4 results in an increase in GM1 levels on the cell membrane (Fig. 3, *A* and *B*). This observation implies that both APOE3 and APOE4 actively regulate GM1 transportation by facilitating the cellular uptake of GM1 lipid structures, subsequently influencing the composition of GM1 on the cell membrane. To visualize GM1 localization and quantify its levels, we employed confocal microscopy (Figs. 3, *B*–*D* and S6). GM1 was labeled using CTSB 555, and the images were quantified using ImageJ. These findings were consistent with SpectraMax data, further confirming the significant increase in GM1 levels within PC-12 cells in the presence of APOE3 and APOE4. In contrast, for HEK-293 cells, the presence of APOE3 and APOE4 did not affect the GM1 levels on the cell membrane (Fig. 3*D*). Confocal microscopy further corroborated these results, showing no significant changes in GM1 levels with or without APOE (Figs. 3*D* and S7). This finding suggests the possibility that APOE plays a role in facilitating the transport and incorporation of GM1 into the cell membrane, and, in turn, may have downstream effects on cellular functions associated with GM1, including intercellular communication, signaling processes, and the promotion of Aβ aggregation. And this process occurs in a cell-type-dependent manner.

To further investigate the cellular uptake process, we labeled APOE with an APOE antibody conjugated to Alexa Fluor 488 to examine its localization relative to GM1 after uptake. As shown in Figure 3*C*, after cellular uptake, APOE exhibited a broad distribution throughout the cell, while GM1 was dispersed across various intracellular regions. However, no significant colocalization was observed. This suggests that the cellular environment and enzymatic processes may dissociate APOE-GM1 lipoproteins. APOE likely traffics through endosomes or lysosomes, while GM1 predominantly associates with membrane dynamics, targeting the plasma membrane or Golgi apparatus.

APOE exhibits superior binding affinity to GM1 compared to cholesterol, suggesting that APOE-GM1 lipoprotein would compete more effectively than APOE-cholesterol lipoprotein for cellular uptake. We separately incubated DiD-labeled APOE-cholesterol lipoprotein and DiD-labeled APOE-GM1 lipoprotein with cells and assessed cellular uptake. We observed a higher cellular uptake of DiD-labeled APOE-GM1 lipoprotein (Fig. 3*E*). We then incubated U-87 MG cells with APOE3 and APOE4-enriched cholesterol lipoproteins, as well as a combination of APOE3 and APOE4-enriched cholesterol lipoproteins and APOE3 and APOE4-enriched GM1 lipoproteins and measured the cholesterol levels on the cells. In the presence of APOE-GM1 lipoprotein, the cellular uptake of APOE-cholesterol lipoprotein decreased (Fig. 3*E*). This observation suggests a competitive relationship between APOE-GM1 lipoprotein and APOE-cholesterol lipoprotein during the uptake process, with cells displaying a greater propensity for the uptake of APOE-GM1 lipoproteins, suggesting the broader functions of APOE in lipid metabolism and cellular homeostasis. Understanding the competitive dynamics between APOE-GM1 and APOE-cholesterol lipoproteins could inform targeted therapies to restore lipid homeostasis and improve cellular function.

This research may elucidate the mechanisms underlying lipid transport within cellular environments, particularly concerning neurodegenerative disorders such as AD, where lipid dysregulation is frequently noted. Given that APOE serves as a principal lipid carrier in cholesterol metabolism and is significantly correlated with the risk of AD, a comprehensive understanding of the competitive interactions between APOE-GM1 and APOE-cholesterol lipoproteins could facilitate the development of targeted therapeutic strategies aimed at modulating lipid homeostasis and enhancing cellular functionality in AD and other neurodegenerative conditions.

### What affects the APOE transportation process?

#### Cell-type dependent expression of APOE receptors

**Figure 4:**
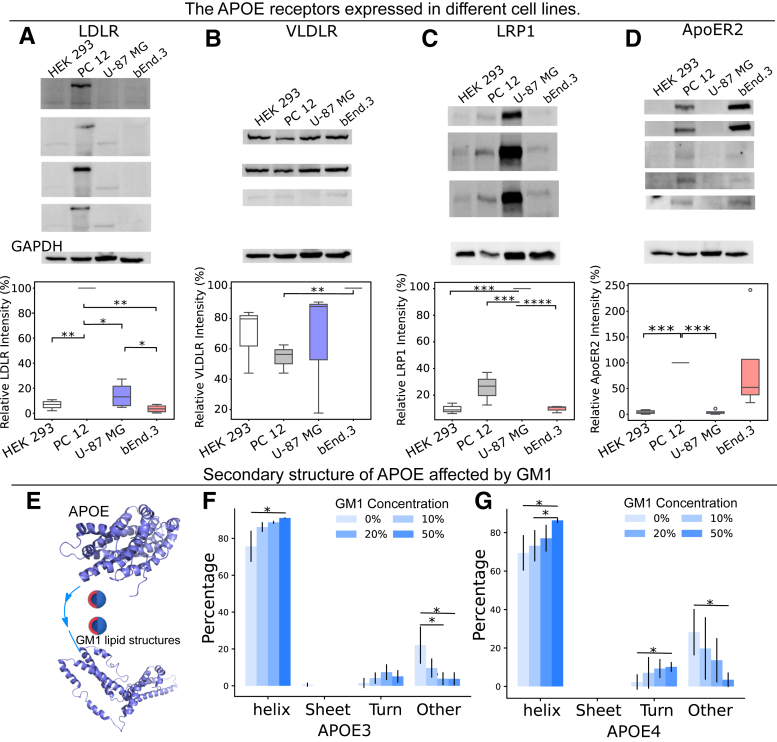
**Western blot results of APOE receptors: LDLR, VLDLR, LRP1, and ApoER2 on differentiated PC-12 cells, U-87 MG, bEnd.3, and HEK-293 cells.***A*, the expression of LDLR on differentiated PC-12 cells, U-87 MG, bEnd.3, and HEK-293 cells (n = 4). *B*, the expression of VLDLR on differentiated PC-12 cells, U-87 MG, bEnd.3, and HEK-293 cells (n = 4). *C*, the expression of LRP1 on differentiated PC-12 cells, U-87 MG, bEnd.3, and HEK-293 cells (n = 3). *D*, the expression of ApoER2 on differentiated PC-12 cells, U-87 MG, bEnd.3, and HEK-293 cells (n = 3). *E*, schematic illustration of determining the changes of APOE secondary structures. *F*, the CD results of APOE 3 under the effect of different GM1 content on the lipid structures (n ≥ 3). *G*, the CD results of APOE 4 under the effect of different GM1 content on the lipid structures (n = 3). *p* value: ns (0.05 < *p* ≤ 1), ∗ (0.01 < *p* ≤ 0.05, ∗∗ (0.001 < *p* ≤ 0.01, ∗∗∗ (0.0001 < *p* ≤ 0.001, ∗∗∗∗ (*p* ≤ 0.0001). (*E*) is created with BioRender.com. APOE, apolipoprotein E; ApoER2, APOE receptor 2; HEK, human embryonic kidney; LDLR, low-density lipoprotein receptor; LRP1, LRPR-related protein 1; PC, l-α-phosphatidylcholine; VLDLR, very low-density lipoprotein receptor.

ApoE is predominantly synthesized by glial cells in the brain, while its receptors are abundant in neurons (35). These receptors include LDLR, LRPR-related protein 1 (LRP1), very low-density lipoprotein (VLDL) receptor (VLDLR), and APOE receptor 2 (ApoER2) (36). LDLR, a transmembrane receptor, stands out as a major APOE receptor (37, 38). Increasing evidence suggests that these receptors, widely expressed in most neurons of the central nervous system, play crucial roles in brain development and may significantly impact the pathogenesis of AD (35, 39, 40). As we found, APOE facilitates the transport of GM1 to the cell membrane. Since we found that the cellular uptake of GM1 lipid structures is contingent on the cell type, we analyzed the expression of APOE receptors, including LDLR, LRP1, VLDLR, ApoER2, and APOE on differentiated PC-12 cells, U-87 MG, bEnd.3, and HEK-293 cells (Figs. 4, *A*–*D* and S8). We find that differentiated PC-12 cells express significantly higher levels of LDLR and ApoER2, U-87 MG cells exhibit significantly higher levels of LRP1, and bEnd.3 cells display significantly higher levels of VLDLR (Fig. 4, *A*–*D*). Differentiated PC-12 cells express the highest overall amount of APOE receptors, whereas HEK-293 cells express the lowest. There is a positive correlation between the expression levels of APOE receptors and the cellular uptake of GM1 lipid structures, with differentiated PC-12 cells showing the highest uptake and HEK-293 cells showing the lowest. This observation implies that the cellular uptake of APOE-delivered GM1 is influenced by the quantity of APOE receptors expressed in the cell.

#### APOE structural changes upon lipid binding

It is well-established that the interaction between APOE and lipids leads to conformational changes in APOE (41, 42). To understand factors influencing the APOE transportation process, we studied whether GM1 binding alters the structural arrangement of APOE, subsequently influencing its recognition by receptors. We characterized the APOE-induced structural changes induced by lipid structures with varying GM1 concentrations by using CD. We incubated GM1 lipid structures with APOE3/APOE4 overnight at 37 °C to generate APOE-enriched lipoproteins. We then assessed the secondary structure using CD (Figs. 4, *E*–*G* and S9). We find that, for both APOE3 and APOE4, an increase in GM1 levels within the lipid structures is associated with higher levels of APOE's helix structure and a reduction in APOE’s other structures. These helical structures play a crucial role in lipid binding (41). GM1 further enhances this interaction by stabilizing the helical conformation of APOE, thereby improving its functional roles in lipid transport and receptor binding, with potential implications for lipid metabolism. We also run DMD simulations for the GM1-bound form of APOE, and we found that during the simulation GM1 completely changed the conformation of APOE. The N terminus and C terminus of APOE extended outward like two arms, grasping the two hydrophobic lipid chains of GM1 (Fig. S13). This finding aligns with our previous experiments, which showed that an increase in GM1 levels within the lipid structure boosts the binding affinity between the lipid structure and APOE.

#### Binding affinity between APOE and LDLR in the presence of GM1

**Figure 5:**
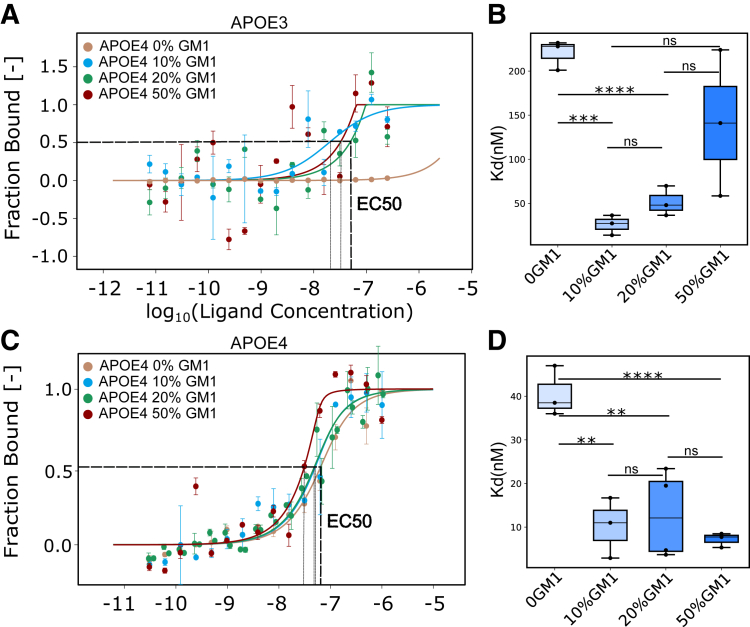
**The binding affinity of APOE-enriched lipoprotein and APOE receptor LDLR using MST.***A* and *B*, the binding affinity between APOE3-enriched lipoprotein with varying GM1 concentration to LDLR. (n = 3) (*C* and *D*) the binding affinity between APOE4-enriched lipoprotein varying GM1 concentration to LDLR (n = 3). *p* value: ns (0.05 < *p* ≤ 1), ∗ (0.01 < *p* ≤ 0.05, ∗∗ (0.001 < *p* ≤ 0.01, ∗∗∗ (0.0001 < *p* ≤ 0.001, ∗∗∗∗ (*p* ≤ 0.0001). APOE, apolipoprotein E; LDLR, low-density lipoprotein receptor; MST, microscale thermophoresis.

The primary APOE receptors that engage in interactions with APOE, facilitating its role in lipid metabolism and transport, include LRP1, LDLR, APOER2, and VLDLR. LDLR, extensively studied as one of the key APOE receptors and widely expressed in various cell types in the human brain, especially in excitatory neurons, plays a central role in regulating LDL cholesterol levels in the bloodstream (43, 44). APOE’s extensive research history underscores its critical importance in maintaining cholesterol homeostasis (45). Lipidation is required for APOE to bind LDLR. To determine the impact of GM1 content on the binding of APOE to its receptors, we labeled APOE3 and APOE4 with RED-NHS and subsequently incubated them with lipid structures containing varying GM1 levels overnight at 37 °C, resulting in the formation of APOE3-lipoprotein and APOE4-lipoprotein. Using MST, we determined the binding affinity between LDLR and APOE3-lipoprotein, as well as APOE4-lipoprotein, across different GM1 concentrations. We find that, for both APOE3 and APOE4, in comparison to the control, where there are no GM1-containing lipid structures, the presence of GM1 reduces the K_D_ value (Fig. 5), thus suggesting that the presence of GM1 enhances the binding affinity of APOE for LDLR.

In summary, the levels of GM1 within lipid structures induce significant alterations in the secondary structure of APOE, thereby enhancing the binding affinity between APOE and its receptor, LDLR. The expression of APOE receptors, along with GM1 levels within lipid structures, plays a crucial role in the regulatory transport process mediated by APOE. Furthermore, we conducted a comparative analysis of the effects of lipid structures containing 20% GM1 *versus* 20% cholesterol on the binding affinity of APOE to LDLR, as illustrated in Fig. S10. Our findings indicate that in the presence of cholesterol, APOE demonstrates a higher K_D_ in comparison to GM1, particularly for the APOE4 variant, suggesting that GM1 more effectively enhances the binding of APOE to LDLR. These results highlight the distinct roles of GM1 and cholesterol in modulating APOE-LDLR interactions.

### The distribution of GM1 on the membrane

**Figure 6:**
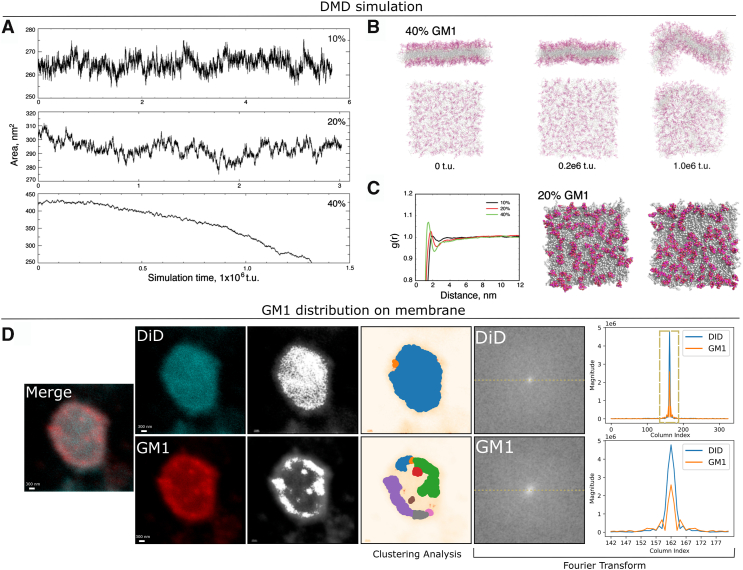
**DMD simulations of POPC membranes mixed with different content of GM1: 10%, 20%, and 40%.** Ten independent simulations, initiated with varying atomic velocities, were conducted for each molecular system. *A*, representative trajectories illustrating the time evolution of the membrane area in the x-y plane indicated that membranes containing 10% and 20% GM1 exhibited stability and preserved their structural integrity throughout the simulations. The DMD time unit (t.u.) is approximately equivalent to 50 fs. *B*, conversely, the membrane with 40% GM1 demonstrated instability, undergoing rapid deformation as evidenced by snapshots captured at various time points along the trajectory. Two perspectives (normal to the *z*-axis and in the *x*-*y* plane) are presented. Lipid tails are depicted in *gray*, while GM1 heads are shown in *red*. *C*, the pair correlation function, g(r), was derived from simulations with differing GM1 ratios. In the case of 40% GM1, the initial metastable phase preceding membrane deformation was utilized for calculations. Each GM1 lipid was represented by a central carbon atom in the head. Despite its charged nature, GM1 exhibited a tendency to interact with itself, with g(r) displaying a peak at short inter-GM1 distances of approximately 1.6 to 3 nm. As GM1 content increased, this pattern became more pronounced, indicating the formation of ordered structures. Two representative snapshots of the membrane with 20% GM1, shown to the *right*, illustrate the self-assembly of GM1 lipids into stipe-like clusters. *D*, a representative image highlights the distribution of lipids and GM1 within the lipid membrane structure. All lipids were labeled with DiD, while GM1 was labeled with CTSB-555. The merged confocal images of DiD and GM1 (column 1), individual images of DiD and GM1 (column 2), *gray*scale representations (column 3), pixel clusters (column 4), 2D Fourier transforms (column 5), and 1D Fourier transforms (column 6) are presented. The 1D Fourier transform illustrates the central horizontal cross-section of the 2D Fourier transform plot. The lower 1D Fourier transform plot corresponds to the region outlined by the dashed box in the upper 1D Fourier transform plot. CTSB-555, Cholera Toxin Subunit B Alexa Fluor 555 Conjugate; DMD, discrete molecular dynamics; POPC, 1-palmitoyl-2-oleoyl-sn-glycero-3-phosphocholine.

GM1 and other gangliosides, primarily studied for their essential intracellular functions, are most extensively investigated as components of the plasma membrane, particularly in neurons where they serve as the predominant sialoglycans (46). Within the lipid bilayer, the distinctive structure of GM1 diminishes the fluidity of the plasma membrane and leads to the retention and enrichment of this ganglioside in specific membrane domains, known as lipid rafts (18). To understand the distribution of GM1 and the influence of elevated GM1 content on membrane morphology and stability, we conducted experiments to evaluate the cellular uptake of lipid structures with varying GM1 concentrations (0%, 10% GM1, 20% GM1, 50% GM1, and 100% GM1) in the presence of APOE and employing the PC-12 cell line. A significant improvement in cellular uptake was observed when the GM1 content in the lipid structure was 20%. To understand the reason for this enhanced cellular uptake at 20% GM1, we performed DMD (47, 48, 49, 50) simulations of 1-palmitoyl-2-oleoyl-sn-glycero-3-phosphocholine (POPC) membrane mixed with 10%, 20%, and 40% GM1 (Fig. 6). To reach time scales associated with the lipid/membrane dynamics (the diffusion coefficient of lipids in the membrane, *D* ∼1 μm^2^/s), we adopted a coarse-grained (CG) lipid model with implicit solvent in DMD simulations. GM1 was modeled as a hybrid all-atom head with CG tails because the interactions of the solvent-exposed heads are important in their self-assembly dynamics (Experimental procedures). With a constant zero-tension in the x-y plane and a constant temperature of 300 K, the membrane area projected to the plane fluctuated (Fig. 6*A*). For membranes with 10% and 20% GM1, all independent simulations reached their steady states during simulations (Fig. S12). With an average area per lipid of ∼0.63 nm^2^, a POPC membrane with 400 lipids per leaflet has an average area of ∼252 nm^2^. The average area of membranes containing both 10% and 20% GM1 was observed to be greater than that of pure POPC membranes. This phenomenon can be attributed to the bulky head structure of GM1 and its net negative charge of -e, which leads to both steric and electrostatic repulsion among GM1 lipids. Consequently, an increase in the ratio of GM1 correlates with an expansion of membrane areas. For the membrane with 40% GM1, the initial membrane area in the x-y plane was significantly higher than in the other two cases, but the large membrane area was not sustainable and rapidly decreased after a short period of simulation time in all independent simulations. With a larger surface area but the same amount of lipids, the membrane thickness is expected to be stretched thin, and thus the membrane experiences high strain. Examination of snapshots along the simulation trajectories revealed that the membrane underwent rapid deformation and started to crumble (Fig. 6*B*), suggesting that the membrane with such a high amount of GM1 was unstable.

To capture the distribution of GM1 on the membrane, we computed the pair correlation function, g(r), of GM1 lipids (Fig. 6*C*). Here, g(r) denotes the average density of other GM1 lipids within a spherical shell with radii between r and r + dr away from each GM1, normalized by the bulk density of GM1. For the case of 40% GM1, we utilized the initial metastable phase before the deformation of membranes in our calculations. The pair correlation function exhibited peaks and valleys at a short distance of approximately 1.6 to 3 nm, indicating the formation of ordered structures. At longer distances, g(r) approached unity. As we increased GM1 ratios, the peaks and valleys became more distinct at shorter distances. Since the membrane with 40% GM1 ultimately proved unstable, we examined GM1 with 20% for structural patterns (Fig. 6*C*). Notably, GM1 on the surfaces formed stripe-like clusters. The interaction likely resulted from hydrogen bonds between hydroxyl groups abundant in the periphery of the head group, while the single carboxyl group positioned itself near the center. In summary, our DMD simulations indicate that a POPC membrane with 20% GM1 maintains membrane integrity while forming stripe-like clusters on the surface, thereby enhancing interactions with APOE.

To elucidate the distribution of GM1 in lipid structures, we implemented a dual-labeling approach, utilizing DiD for all lipids on lipid structures and CTSB-555 for GM1 on lipid structures. Subsequently, we used confocal microscopy to analyze the spatial arrangement of lipids. We converted the confocal images to grayscale, revealing a nonuniform distribution of GM1, in contrast to the more homogeneous distribution observed for other lipids labeled by DiD in the system (Figs. 6*D* and S11). To address potential biases introduced by faint pixel intensities and ensure accurate observations, we applied the DBSCAN algorithm (51) directly to the pixel clustering in the images. The clustering results indicated that pixels representing DiD were grouped into a single class, while those representing GM1 were distributed across multiple classes. Given that DBSCAN classifies data points based on density, this further suggests that DiD's distribution is uniform, whereas GM1's distribution is highly nonuniform. In addition, we performed a Fourier analysis of the confocal images. The analysis revealed that DiD's spectrum exhibited a highly intense central peak, typically indicative of a uniform pixel distribution. In contrast, GM1's spectrum displayed a lower central peak with smaller frequencies around it, indicating a more uneven distribution compared to DiD. Hence, both cluster analysis and Fourier analysis collectively emphasize the highly nonuniform distribution of GM1 on lipid structures, underscoring the importance of accounting for biases associated with faint pixel intensities during visual interpretation. Our experimental observations are in concert with DMD simulations.

In summary, we observed that membranes containing an excessive amount of GM1 (40%) exhibited instability, whereas those with 20% GM1 preserved their integrity and formed stripe-like clusters on the surface. These GM1 clusters may enhance the cellular uptake of lipid structures under the regulation of APOE, as previously discussed.

## Discussion

The ε4 allele of the APOE gene has long been recognized as the strongest genetic risk factor for AD (6, 39). Despite multiple studies linking APOE to neuronal membrane integrity (52), Aβ clearance (6), and neuroinflammation (53), the precise mechanisms underlying its role in AD pathogenesis remain poorly understood (54). Our study reveals a novel biological role for APOE in mediating the transport of GM1, thereby establishing a mechanistic link between APOE function, lipid homeostasis, and AD pathogenesis.

Previous studies have highlighted the importance of GM1 in AD etiology, particularly its role in promoting Aβ aggregation (8, 23, 24, 55, 56, 57). Here, we determine whether APOE acts as a transporter of GM1. Using MST, we show that APOE binds GM1 with greater affinity than cholesterol, indicating a competitive relationship between these two lipids for APOE-mediated transport. This dual lipid transport capability positions APOE as a key regulator of membrane lipid composition and provides a potential mechanism through which altered lipid environments contribute to AD pathology. Our findings suggest that GM1 enrichment on neuronal membranes may disrupt cholesterol homeostasis by outcompeting cholesterol for APOE binding. Moreover, APOE-mediated alterations in GM1 levels could interfere with lipid raft stability, thereby potentially impacting signal transduction pathways such as Reelin signaling, which plays crucial roles in synaptic plasticity and neuronal development.

Cellular experiments further support the role of APOE in facilitating GM1 transport in a cell-type-specific manner. Since APOE receptor expression varies among brain cell types and is particularly abundant in neurons (35), the extent of GM1 uptake is likely influenced by the differential expression of receptors. In neurons with high APOE receptor expression, this mechanism may lead to substantial changes in membrane lipid composition and signaling activity, potentially contributing to disease progression. In contrast, cell types with low APOE receptor expression may remain relatively unaffected.

Notably, we observed robust GM1 uptake in bEnd.3 cells, which are brain endothelial cells used as a model of the blood–brain barrier (34), suggesting that APOE may regulate GM1 transport across the blood–brain barrier. Given GM1's higher affinity for APOE compared with that of cholesterol, GM1-containing lipid structures could potentially be harnessed as APOE-regulated carriers for drug delivery into the brain. Supporting this idea, previous reports have identified lipid-free APOE and GM1-enriched exosomes in cerebrospinal fluid (58). We propose that APOE may facilitate the transport of GM1-rich exosomes in cerebrospinal fluid, influencing lipid and protein distribution in the central nervous system.

To better understand the structural basis of GM1 transport, we combined CD, confocal imaging, and DMD simulations. We found that membranes with 20% GM1 retained their structural integrity and formed stripe-like GM1 clusters, changing the secondary structure of APOE and enhancing cellular uptake in the presence of APOE. Interestingly, the binding of APOE to LDLR appeared more variable in the presence of GM1, particularly for APOE4, suggesting that GM1 distribution on the membrane may influence APOE-LDLR interactions.

Collectively, our findings reveal a previously unrecognized mechanism by which APOE regulates GM1 distribution, thereby promoting Aβ oligomerization and aggregation. This work sheds new light on lipid-mediated dysfunctions that may underlie APOE-driven AD pathogenesis.

## Experimental procedures

### Lipid structures and APOE-enriched lipoproteins preparation

We dissolved phosphatidylcholine (PC, Sigma-Aldrich), sphingomyelin (VWR International), cholesterol (Thermo Fisher Scientific), and GM1 (Sigma-Aldrich) in a chloroform/methanol (1:1, V:V) mixture at a total concentration of 5 mM. Various lipid structures with different compositions were prepared to examine their impact, including 50% PC + 50% SM; 45% PC + 45% SM + 10% GM1; 40% PC + 40% SM + 20% GM1; 25% PC + 25% SM + 50% GM1; and 40% PC + 40% SM + 20% cholesterol. We then dried the mixed solution under vacuum at room temperature and rehydrated the lipid film in PBS buffer (pH 7.4) with a 1 mM lipid concentration at 42 °C. After vortexing at least four times for 1 h, we sonicated the turbid emulsion for 1 h. In addition, to assess the effect of lipid structure size, we prepared lipid structures by sonicating the rehydrated suspension for 15 min, followed by five cycles of freeze-thawing in liquid nitrogen and a 60 °C water bath. Subsequently, we extruded the resulting suspension 16 times using an Avanti extruder with 800 nm pore-size polycarbonate filters for lipid structure filtration. The size distribution of the lipid structures was determined using dynamic light scattering.

We incubated recombinant human APOE3 or APOE4 (Peprotech) with lipid structures (total lipid concentration for the lipid structure: 200 μM) at a concentration of 47.6 μg/ml in a water bath at 37 °C overnight to form APOE-enriched lipoproteins.

### Assessment of the binding affinity between lipid structures to APOE

We adhered to the manufacturer's instructions to label APOE3 and APOE4 with the Monolith protein labeling kit RED-NHS 2nd generation (NanoTemper Technologies). We mixed 90 μl of 10 μM APOE3 or APOE4 dissolved in PBS with 10 μl of 300 μM RED-NHS 2nd dye by pipetting up and down. We then incubated the mixture for 30 min at room temperature in the dark and purified the labeled APOE stock solution by removing the excess dye using a column. Following the manufacturer's guidelines, we employed MST to evaluate the binding affinity of lipid structures with varying GM1 concentrations to APOE3 and APOE4.

We added 10 μl of the diluted APOE solution at a concentration of 50 nM, following the MST manufacturer's instructions, into each of the tubes numbered 2 to 16 in sequence, excluding tube number 1. We added 20 μl of lipid structures to the first sample tube, then transferred 10 μl to the next tube, mixing it with the APOE solution. We started with stored lipid structures at a total lipid concentration of 1 mM and repeated this dilution for the remaining tubes. We discarded the 10 μl excess from tube 16. The 16 capillary tubes (NanoTemper Technologies) were inserted into each sample tube to facilitate sample entry into the capillary, and the capillaries were sequenced and detected using MST.

### Negative staining of lipid structures

We adsorbed lipid structures to glow-charged carbon-coated 400-mesh copper grids for 2 min and stained with 2% (wt/vol) uranyl acetate in water. Transmission electron microscopy images were taken using a FEI Tecnai 12 transmission electron microscope (Thermo Fisher Scientific) at 80 kV. Images were captured on a Gatan RIO camera (4k × 4k pixels) using the Digital Micrograph software (Gatan).

### Uptake of lipid structures by cells

We obtained PC-12 cells from the American Type Culture Collection (ATCC) and culture and differentiated them according to previously published methods (24). We obtained HEK-293 cells from Jong Yun Lab (Penn State College of Medicine) and cultured them in Dulbecco's modified Eagle's medium (DMEM) (VWR International) supplemented with 10% fetal bovine serum (Thermo Fisher Scientific), 1% nonessential amino acids (Thermo Fisher Scientific), and 1% Penicillin/Streptomycin (Thermo Fisher Scientific) at 37 °C with 5% CO_2_. Similarly, we obtained bEnd.3 cells from the ATCC and cultured in DMEM supplemented with 10% fetal bovine serum and 1% Penicillin/Streptomycin at 37 °C with 5% CO_2_. We obtained U-87 MG cells from Jeffrey Neighbors lab (Penn State College of Medicine) and cultured them in Eagle's Minimum Essential Medium (ATCC) supplemented with 10% fetal bovine serum and 1% Penicillin/Streptomycin at 37 °C with 5% CO_2_.

We labeled 500 μl of 1 mM lipid structures with different contents by adding 10 μl of 1 mM DiI Stain (1,1′-dioctadecyl-3,3,3′,3′ tetramethylindocarbocyanine perchlorate) (Thermo Fisher Scientific) or 10 μl of 1 mg/ml Invitrogen DiD oil (DiD, Thermo Fisher Scientific) to the lipid solution during preparation. We incubated DiI or DiD-labeled 0.5 mM lipid structures with 47.6 μg/ml APOE3 or APOE4 at 37 °C overnight to form APOE-enriched lipoproteins. To investigate the impact of GM1 content on cellular uptake, we incubated differentiated PC-12 cells with various 15 μM lipid structures (50% PC + 50% SM; 45% PC + 45% SM + 10% GM1; 40% PC + 40% SM + 20% GM1; 25% PC + 25% SM + 50% GM1; and 40% PC + 40% SM + 20% cholesterol), and the correspondingly APOE3 and APOE4 (3.66 μg/ml)-enriched lipoproteins for 4 h, using seeded cells at a density of 2000 cells per well. To characterize the cellular uptake of lipid structures in different cell lines, we added APOE3 or APOE4-enriched lipoproteins (40% PC + 40% SM + 20% GM1) to differentiate PC-12, bEnd.3, U-87 MG, and HEK-293 cells and incubated them for 4 h, using seeded cells at a density of 2000 cells per well. We added equivalent quantities of lipid structures or PBS to the cells as controls for comparison with APOE-enriched lipoproteins. We removed excess DiI and DiD-labeled APOE-enriched lipoproteins from the medium by thoroughly washing with PBS three times. We measured the fluorescence intensity of DiI and DiD using SpectraMax i3 to elucidate the uptake of APOE-enriched lipoproteins. For DiI, we used the excitation wavelength of 522 nm, and the emission wavelength of 560 nm. For DiD, we used the excitation wavelength of 644 nm, and the emission wavelength of 670 nm. Each sample consisted of six wells. We repeated tests taken from the distinct samples.

We assessed the cellular uptake of cholesterol by incubating U-87 MG cells with DiD-labeled APOE-enriched cholesterol lipoproteins (40% PC + 40% SM + 20% cholesterol) or DiD-labeled APOE-enriched GM1 lipoproteins (40% PC + 40% SM + 20% GM1) overnight. Afterward, we removed the excess lipoproteins to determine the fluorescence intensity. To investigate the potential competition between GM1 and cholesterol lipid structures in cellular uptake, we incubated U-87 MG cells with APOE-enriched cholesterol lipoproteins, along with a combination of APOE3 and APOE4 (3.66 μg/ml)-enriched cholesterol lipoproteins and APOE3 and APOE4 (3.66 μg/ml)-enriched GM1 lipoproteins overnight. We determined the changing of cholesterol level on the cell membrane using Amplex Red Cholesterol Assay Kit (Thermo Fisher Scientific) according to the manufacturer's instructions.

To characterize the changes in GM1 levels on the cell membrane, we incubated differentiated PC-12 cells in a 96-well plate with APOE (3.66 μg/ml)-enriched GM1 lipoproteins. After washing to eliminate surplus APOE-enriched GM1 lipoproteins, we added 100 μl of 1 μg/ml CTSB to each well and incubated with cells for 30 min at room temperature to label GM1 on the cell membrane. Then, we remove the excess CTSB by washing it with PBS three times. We measured the fluorescence intensity of CTSB on the cell membrane to characterize the changes in GM1 levels using SpectraMax i3 with an excitation wavelength of 555 nm and an emission wavelength of 575 nm.

We coated the cell culture chamber slide with poly-l-lysine (Bio-Techne) at 37 °C for 30 min and seeded cells at 20,000 cells/well. After incubating cells with DiI- and DiD-labeled APOE (3.66 μg/ml)-enriched lipoproteins overnight, we removed the excess APOE-enriched lipoproteins in the medium by washing with PBS three times. We fixed the cells with 600 μl of 4% paraformaldehyde solution (VWR International) in PBS for 15 min and washed them with PBS three times. We labeled GM1 on the cell membrane with 300 μl of 1 μg/ml CTSB for 30 min at room temperature, followed by washing with PBS three times. We performed nuclear labeling using 600 μl of 2 μg/ml 4′,6-diamidino-2-phenylindole (DAPI, Sigma-Aldrich) for 10 min, and we washed the cells with PBS three times. We labeled APOE by first permeabilizing the cells with 0.3% Triton X-100 in PBS for 7 min at room temperature. Afterward, we washed the cells three times with PBS. We then blocked the cells with 2% bovine serum albumin for 30 min. Next, we added primary APOE recombinant rabbit monoclonal antibody (diluted 1:1000 in 2% bovine serum albumin, Invitrogen) and incubated it for 2 h at room temperature. Following this, we washed the cells three times with PBS. Finally, we added Alexa Fluor 488 goat anti-rabbit antibody as the secondary antibody (diluted 1:1000, Thermo Fisher Scientific) for 2 h. We used confocal microscopy (LEICA SP8 STED 3X) to determine the cellular uptake of the lipid structures and the localization of GM1 and APOE, and analyzed fluorescence intensity using ImageJ.

### Assessing the effect of GM1 levels on the binding affinity of APOE-enriched lipid structures to LDLR

To determine the binding affinity between APOE-lipoprotein and its receptor LDLR (MedChemExpress), we first labeled APOE3 and APOE4 with RED-NHS 2nd generation, as described earlier. Subsequently, we incubated lipid structures with different GM1 concentrations with the labeled APOE at 37 °C overnight to produce APOE-enriched lipoprotein. We then used MST to assess the binding affinity between these 20 nM APOE-enriched lipid structures and 250 nM APOE receptor, LDLR. We repeated each test at least three times.

### Cell lysis and APOE receptor protein quantification

After culturing the differentiated PC-12, bEnd.3, U87-MG, and HEK-293 cells, we added cold RIPA buffer or harsh detergent with 0.2% SDS for LRP1 with Pierce Protease Inhibitor (1 tablet/10 ml, Thermo Fisher Scientific) to the cells and incubated them at 4 °C for 15 min. Subsequently, we scraped and transferred the cell lysate to 1.5 ml tubes and used sonication to break up any remaining cells with one pulse at 45% power on ice. After centrifuging the lysates at 12,000 rpm for 15 min at 4 °C, we discarded the pellet. The concentration of total protein was quantified using a bicinchoninic acid (BCA) kit. To do this, we diluted the obtained total protein from cells 10 times with 10 μl in duplicate wells, added 200 μl of BCA reagent (mix 50:1 A:B), and incubated it for 30 min at 37 °C. The absorption was then determined at 562 nm using SpectraMax i3.

We employed western blot to quantify the levels of APOE receptors, including LRP1, LDLR, APOER2, and VLDLR in different cell lines. The loaded samples were normalized according to BCA quantification results, and the samples were heated at 95 °C for 10 min. An 8% polyacrylamide gel was used for the western blot. We used primary antibodies included recombinant anti-LDL receptor antibody (diluted 1:500, Abcam), anti-LRP1 antibody produced in rabbit (diluted 1:1000, Sigma-Aldrich), anti-ApoER2 antibody (diluted 1:1000, Abcam), anti-VLDLR antibody produced in rabbit (diluted 1:1000, Sigma-Aldrich), and control GAPDH antibody (diluted 1:1000, Cell Signaling Technology). We used anti-rabbit horseradish peroxidase (diluted 1:1000, Thermo Fisher Scientific) as the secondary antibody. We quantified bands using ImageJ.

### Secondary structure

We incubated APOE3 or APOE4 (47.6 μg/ml) in the presence of 1 mM lipid structures containing varying GM1 contents (50% PC + 50% SM; 45% PC + 45% SM + 10% GM1; 40% PC + 40% SM + 20% GM1; 25% PC + 25% SM + 50% GM1). We rehydrated the mixture with 10 mM sodium phosphate buffer overnight in a water bath at 37 °C. We determined the secondary structure characteristics of APOE3 and APOE4 using a Jasco J-1500 circular dichroism spectrophotometer, employing the relative lipid structures solution as the control. We placed the samples in 1 mm path-length (200 μl) cuvettes and recorded the spectra in the range of 185 to 240 nm with a 1 nm data pitch and a scanning speed of 50 nm/min at 25 °C. We averaged three accumulations for each measurement.

### The distribution of GM1 on the membrane

We labeled all lipids on the lipid structures membrane using DiD, as mentioned earlier. Subsequently, we labeled GM1 on the 10% GM1 lipid structures by incubating 50 μl of 100 μg/ml CTSB with 100 μl of lipid structures for 30 min in the dark and removed the excess CTSB by washing with PBS using a 300 kDa MWCO centrifugal filter tube. We coated the microscope slides with poly-l-lysine, and we determined the distribution of GM1 on the lipid structures membrane using a confocal microscope (LEICA SP8 STED 3X).

### DMD simulation

The DMD algorithm serves as a rapid and versatile tool for molecular dynamics simulations. In this study, we modeled the membrane using CG lipids in an implicit solvent environment. We maintained a constant pressure with zero tension during the CG membrane simulations by employing a Berendsen barostat. Each POPC lipid is represented by 15 CG beads, which include one choline bead, one phosphate bead, and one glycerol bead in the head, along with two ester beads that connect 10 hydrophobic beads, forming two linear tails. We derived the intra- and inter-CG lipid interactions self-consistently through an iterative Boltzmann inversion method, which reproduces all pair correlation functions observed in equilibrium all-atom molecular dynamics simulations with explicit solvent at room temperature and ambient pressure. In contrast, we represented GM1 with a hybrid approach in our simulation. The head of the GM1 lipid features an all-atom representation, while its two tails are CG, similarly to the CG POPC lipids. We modeled the interactions of the all-atom GM1 heads using the MedusaScore force field, an extension of the Medusa force field designed for small-molecule ligands, which accurately captures protein-ligand interactions.

Initial structures of three membrane systems with 10%, 20%, and 40% GM1 were generated by CHARMM-GUI. In addition, the initial structure of the GM1-bound form of APOE was also generated by CHARMM-GUI. Each of the membranes was composed of 800 lipids, with 400 in each leaflet. For each molecular system, 10 independent simulations with different initial atomic velocities were performed. With more hybrid GM1 lipids, more calculations were required to reach the same simulations. With 10% GM1, each independent simulation reached ∼5.5 × 10^6^ time units (t.u.), corresponding to ∼270 ns, and the cumulative time was about ∼2.7 μs. The time unit is equivalent to 50 fs. With 20% GM1, each independent simulation lasted ∼3 × 10 (6) t.u., or ∼150 ns, and the cumulative time was ∼1.5 us. For the system with the most GM1, each independent simulation lasted ∼1.4 × 10^6^ t.u., or ∼70 ns, and the cumulative time was ∼0.7 μs. The equilibration of the membrane system in the constant tension DMD simulations could be evaluated from the time evolution of the membrane area in the x-y plane (*e.g.*, Fig. 4). Simulation of GM1-bound form of APOE was run for ∼500 ns.

## Data availability

All data can be accessed upon request from the corresponding author.

## Code Availability

All custom scripts can be accessed upon request from the corresponding author.

## Supporting information

This article contains supporting information.

## Conflict of interest

The authors declare that they have no conflicts of interest with the contents of this article.
