# Supplementary material for: Apolipoprotein E (APOE) regulates the transport of monosialotetrahexosylganglioside (GM1)
Source: J Biol Chem. 2025 Sep 27;301(11):110778. doi: 10.1016/j.jbc.2025.110778 (PMC12605260; doi:10.1016/j.jbc.2025.110778)
Supplement: Supporting Figures [file mmc1.docx]

SUPPLEMENTARY INFORMATION

**APOE regulates the transport of GM1**

Dong Yan Zhang^1^, Jian Wang^1^, Gangtong Huang^2^, Martin Dokholyan^3^, Smaranda Willcox^4^, Jack Griffith^4^, Feng Ding^2^, and Nikolay V. Dokholyan^1,5,6^*

*^1^Department of Neurology, University of Virginia, School of Medicine, Charlottesville, VA, 22903, USA.*

*^2^Department of Physics and Astronomy, Clemson University, Clemson, SC, 29634, USA.*

*^3^Department of Neuroscience and Experimental Therapeutics, Penn State College of Medicine, Hershey, PA, 17033-0850, USA.*

*^4^Lineberger Comprehensive Cancer Center and Departments of Microbiology and Immunology, and Biochemistry and Biophysics, The University of North Carolina at Chapel Hill, Chapel Hill, NC, 27599, USA.*

*^5^Department of Biomedical Engineering, University of Virginia, School of Medicine, Charlottesville, VA, 22903, USA.*

***^6^****Department of Neuroscience, University of Virginia, School of Medicine, Charlottesville, Virginia, USA*

**Corresponding author: Nikolay V. Dokholyan, E-mail:* [*dokh@virginia.edu*](mailto:dokh@virginia.edu)*.*


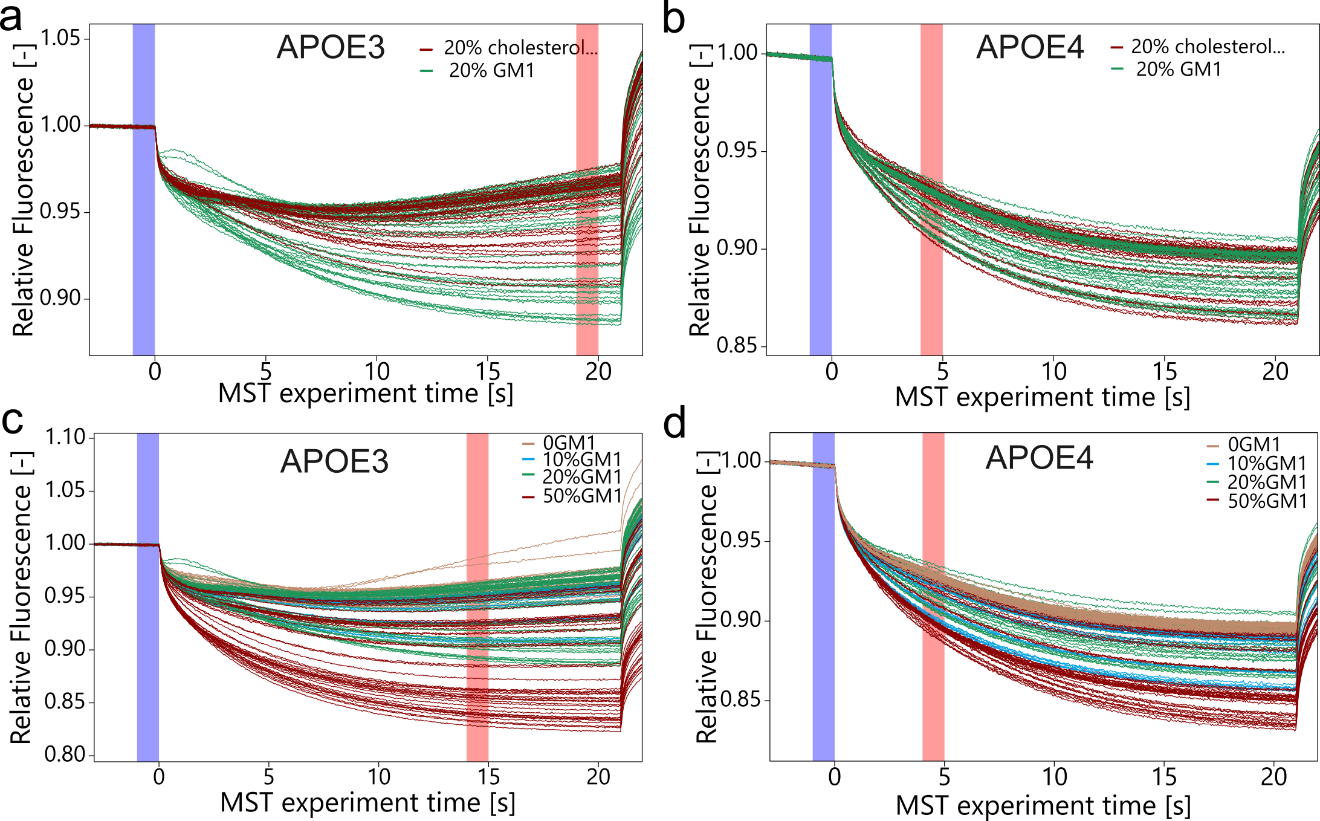


Figure S1. MST traces of measuring the binding affinity between APOE and lipid structures.

(a) MST traces for the binding between APOE3 and lipid structures containing GM1 or cholesterol (n=3). (b) MST traces for the binding between APOE4 and lipid structures containing GM1 or cholesterol (n=3). (c) MST traces for the binding between APOE3 and lipid structures containing GM1 with various concentration (n=3). (d) MST traces for the binding between APOE4 and lipid structures containing GM1 with various concentration (n=3).


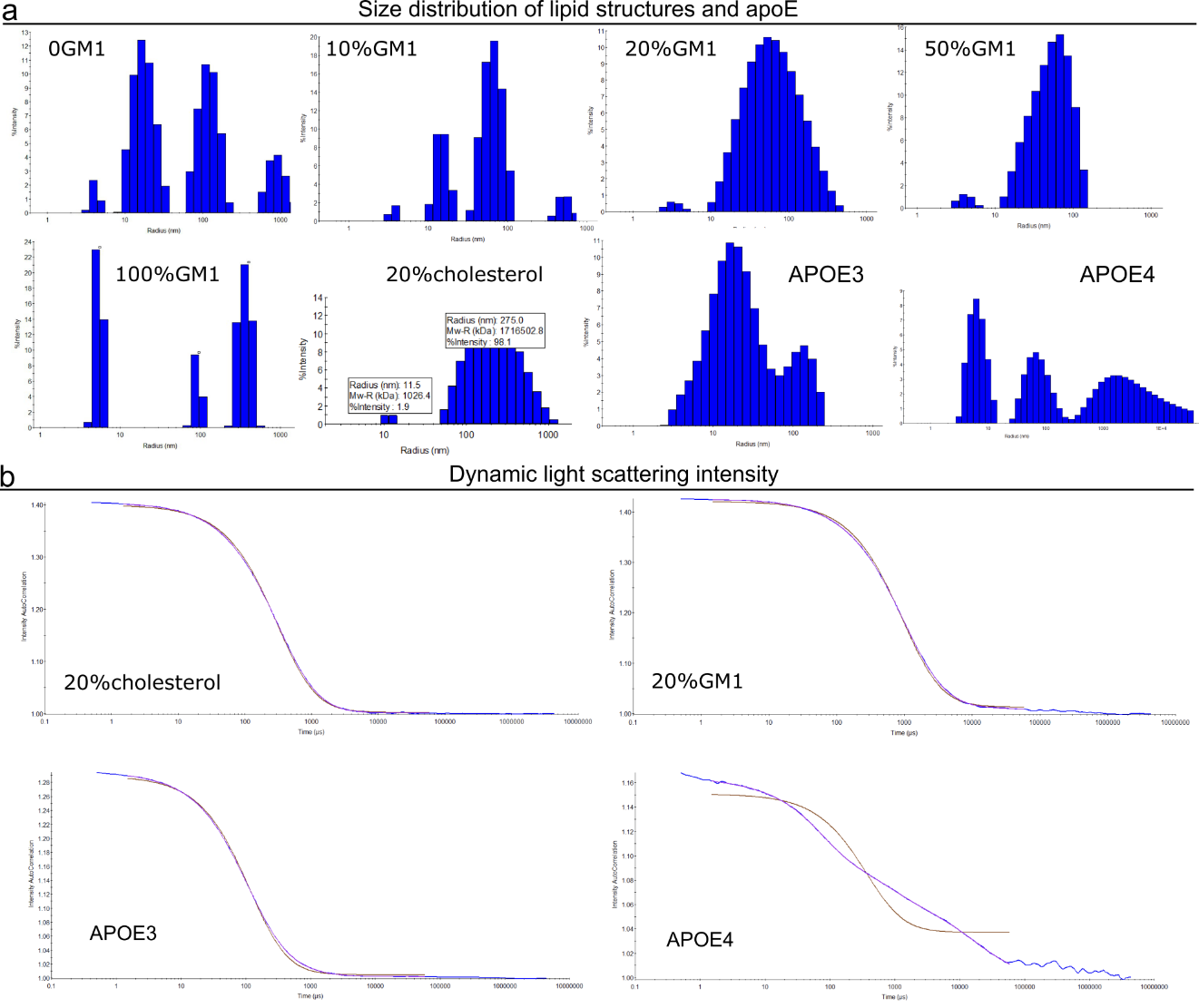


Figure S2. Characterization of lipid structures and APOE.

(a) Dynamic light scattering (DLS) was used to determine the size distribution of lipid structures (1 mM total lipid concentration) and APOE3/APOE4 proteins (47.6 µg/mL in PBS buffer). (b) DLS signal intensity: The signal intensity of lipid structures is higher than that of APOE proteins, reflecting a higher particle concentration in the lipid structure samples compared to the APOE samples.


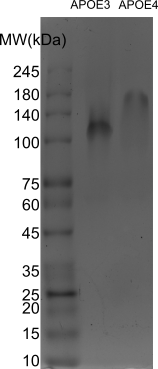


Figure S3. Nondenaturing gel electrophoresis analysis of the APOE3 and APOE4 proteins used in this study.


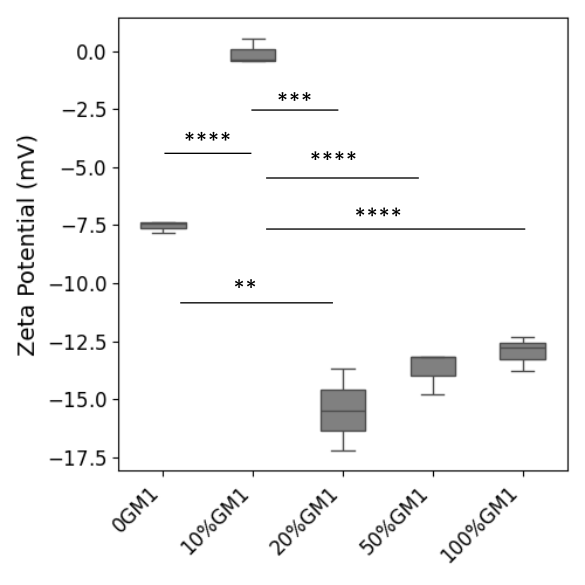


Figure S4. Zeta potential measurement of lipid structures with varying GM1 concentrations. Lipid structures were prepared at a total lipid concentration of 1 mM with varying GM1 concentrations (n = 3). P-value: ns (not significant, 0.05 < p ≤ 1), * (0.01 < p ≤ 0.05), ** (0.001 < p ≤ 0.01), *** (0.0001 < p ≤ 0.001), **** (p ≤ 0.0001).


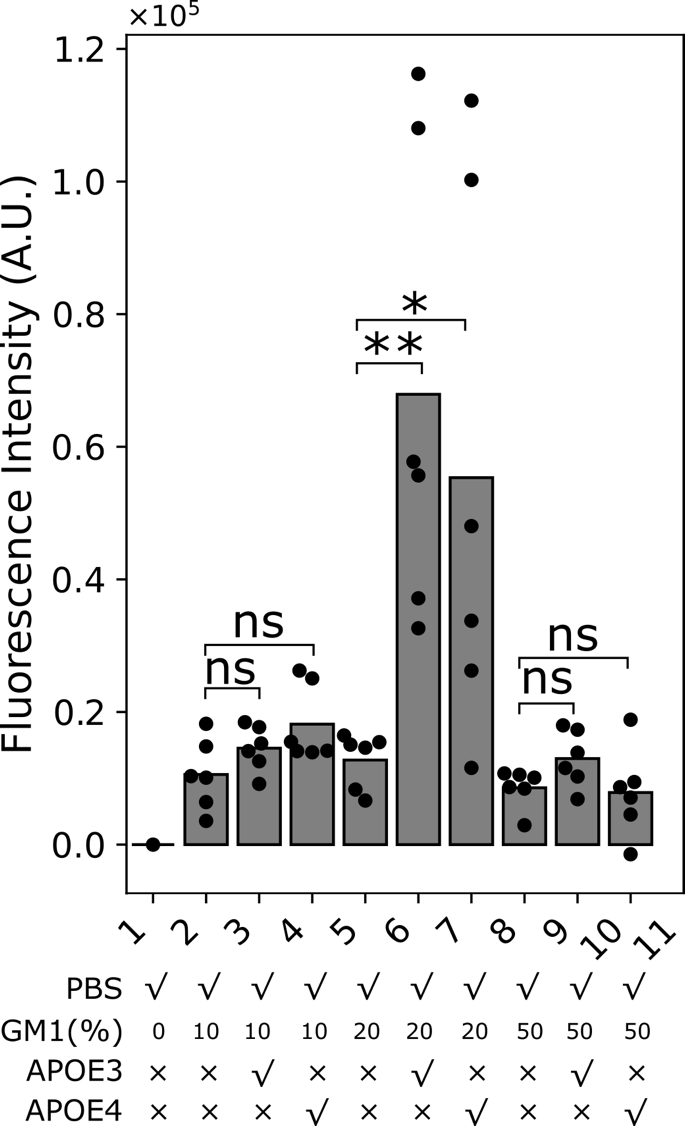


Figure S5. Determination of PC-12 cellular uptake of lipid structures with varying GM1 concentrations. We seeded PC-12 cells in 96-well plates at a density of 2000 cells/well and differentiated PC-12 for 1 week. Subsequently, we incubated DiI-labeled lipid structures with varying GM1 concentrations, along with corresponding APOE3 and APOE4-enriched lipoproteins with cells for 4 hours, and then we determined the cellular uptake. (n>=6) P-value: ns (0.05 < p <= 1), * (0.01 < p <= 0.05, ** (0.001 < p <= 0.01, *** (0.0001 < p <= 0.001, **** (p <= 0.0001).


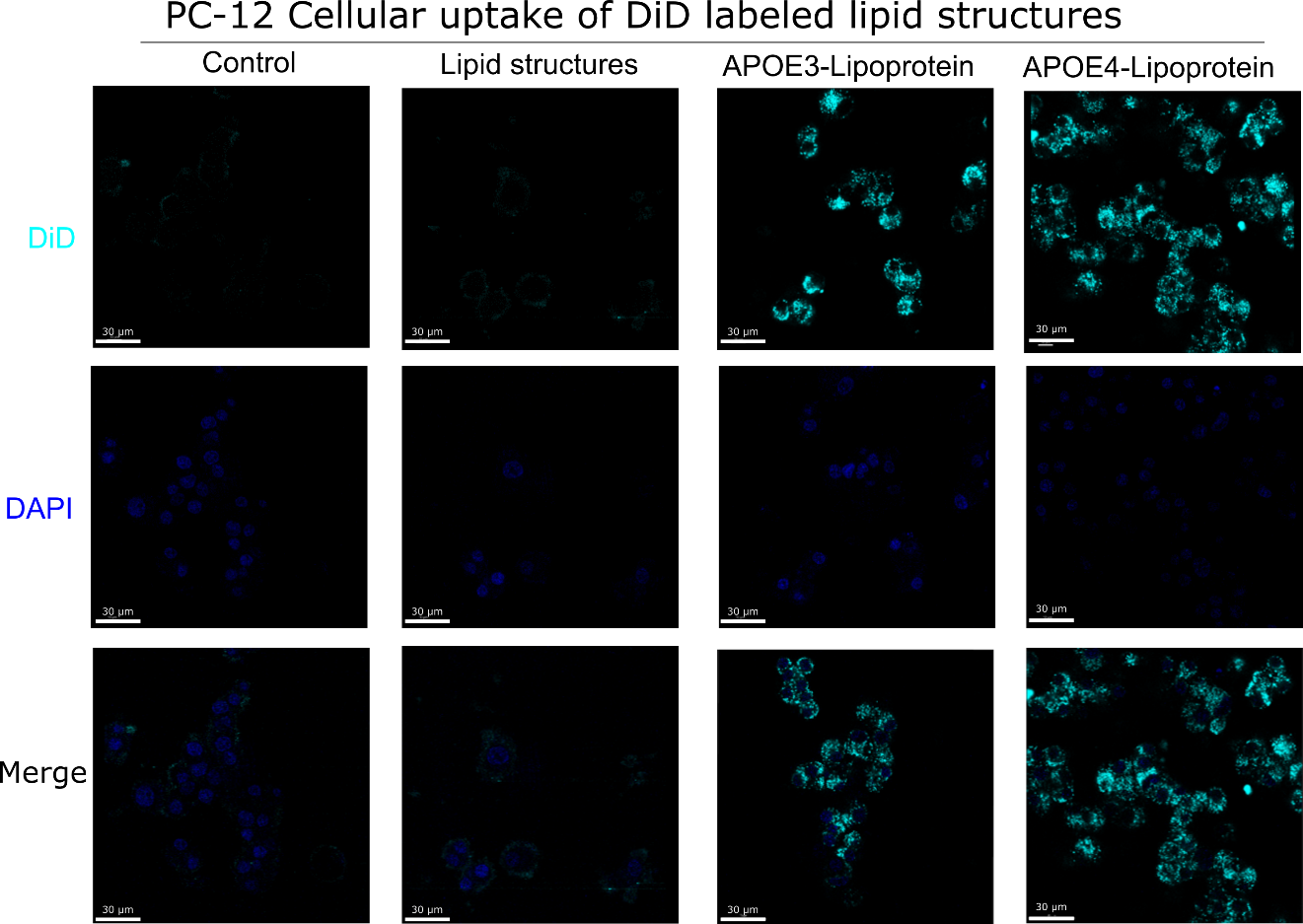


Figure S6. Cellular uptake of DiD-labeled lipid structures on PC-12 cells determined with confocal microscope.


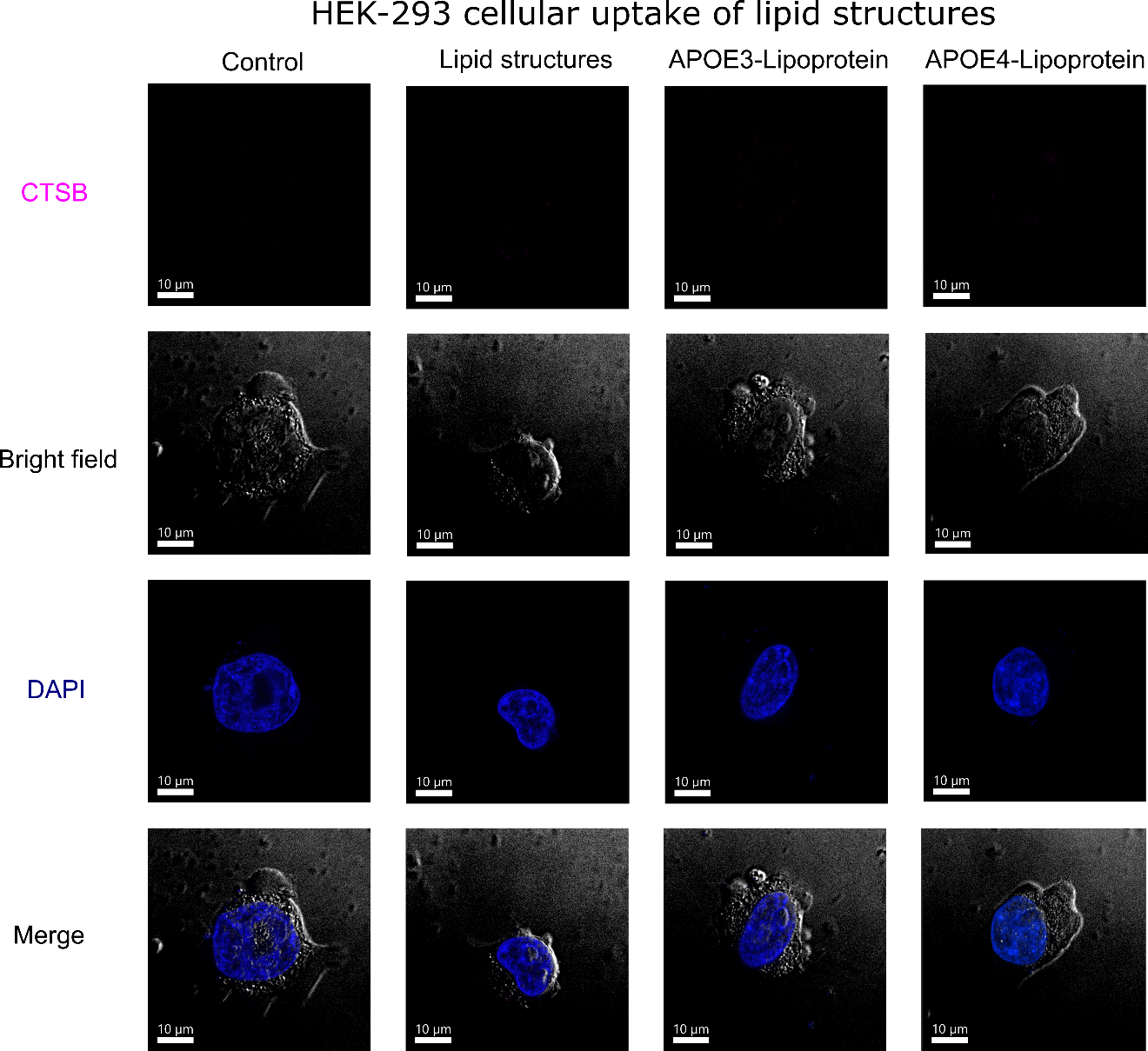


Figure S7. Representative images showing GM1 levels in HEK-293 cells following cellular uptake, as determined by confocal microscopy.


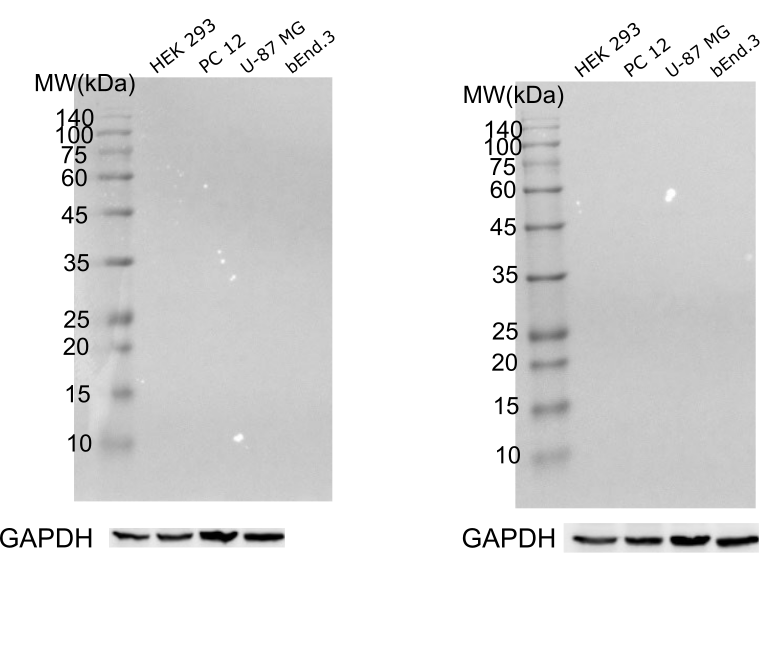


Figure S8. Western blot analysis of APOE expression in various cell lines. In this experiment, we used the same samples (identical in concentration and volume) as those employed for testing the expression levels of APOE receptors.


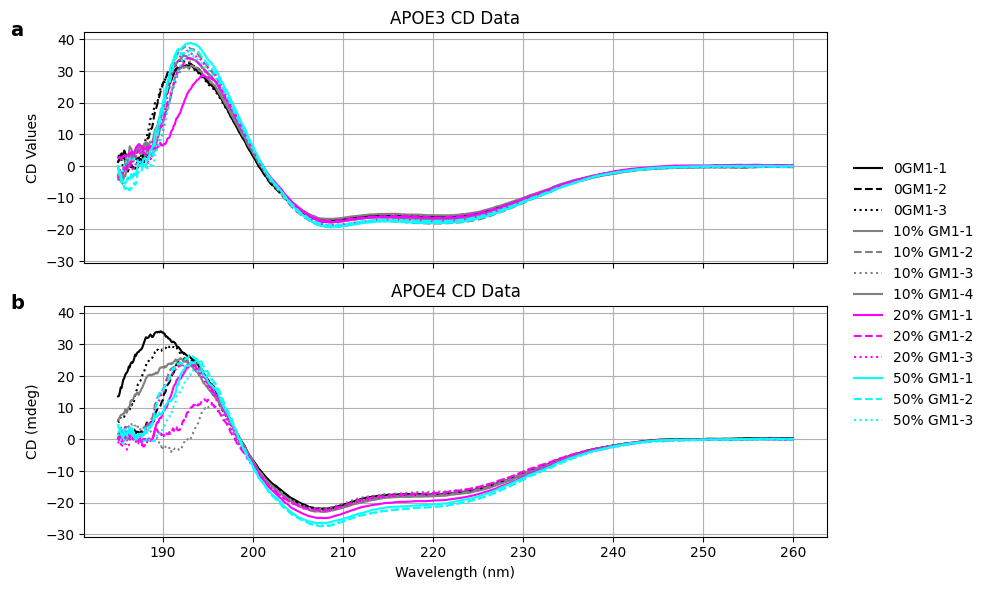


Figure S9. Secondary structures of APOE3 and APOE4 under the effect of varying GM1 concentrations within the lipid structures. (a) CD results of APOE 3 with different GM1 content in the lipid structures. (b) CD results of APOE 4 with different GM1 content in the lipid structures.


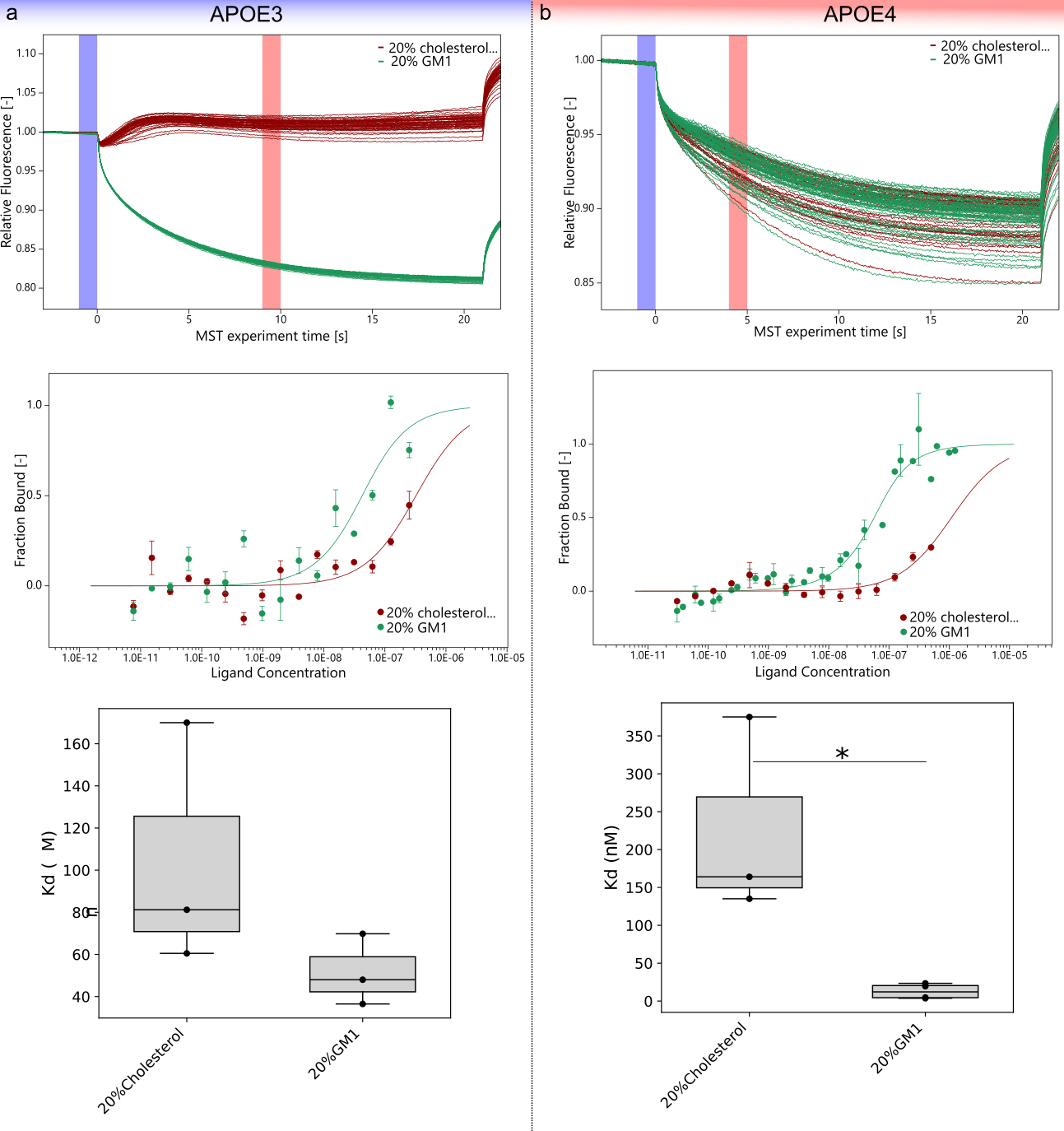


Figure S10. Comparison of the binding affinity between APOE-enriched cholesterol or GM1 lipoproteins and the APOE receptor LDLR using MST. (a) Binding affinity between APOE3-enriched cholesterol or GM1 lipoproteins and LDLR (n = 3). (b) Binding affinity between APOE4-enriched cholesterol or GM1 lipoproteins and LDLR (n = 3). P-value:: ns (not significant, 0.05 < p ≤ 1), * (0.01 < p ≤ 0.05), ** (0.001 < p ≤ 0.01), *** (0.0001 < p ≤ 0.001), **** (p ≤ 0.0001).


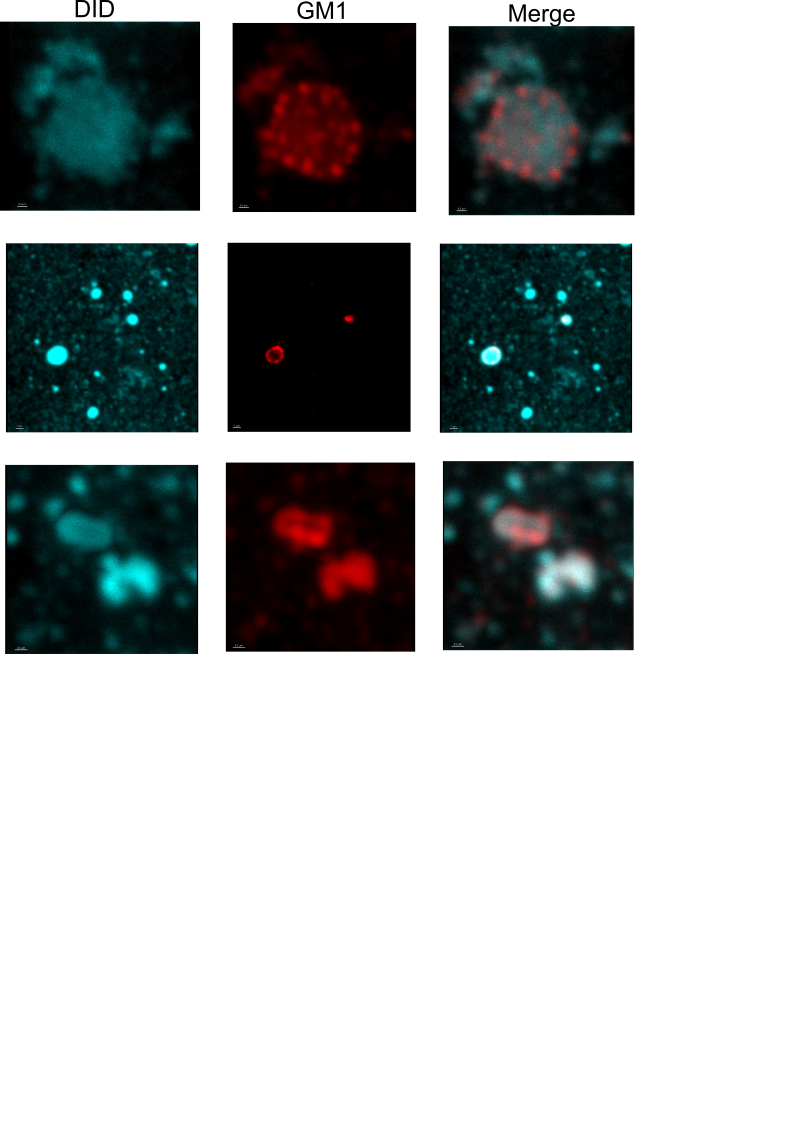


Figure S11. Representative image showcasing the distribution of lipids and GM1 on the lipid structure membrane. All lipids on the lipid structures were labeled with DiD, while GM1 on the lipid structures was labeled with CTSB-555.


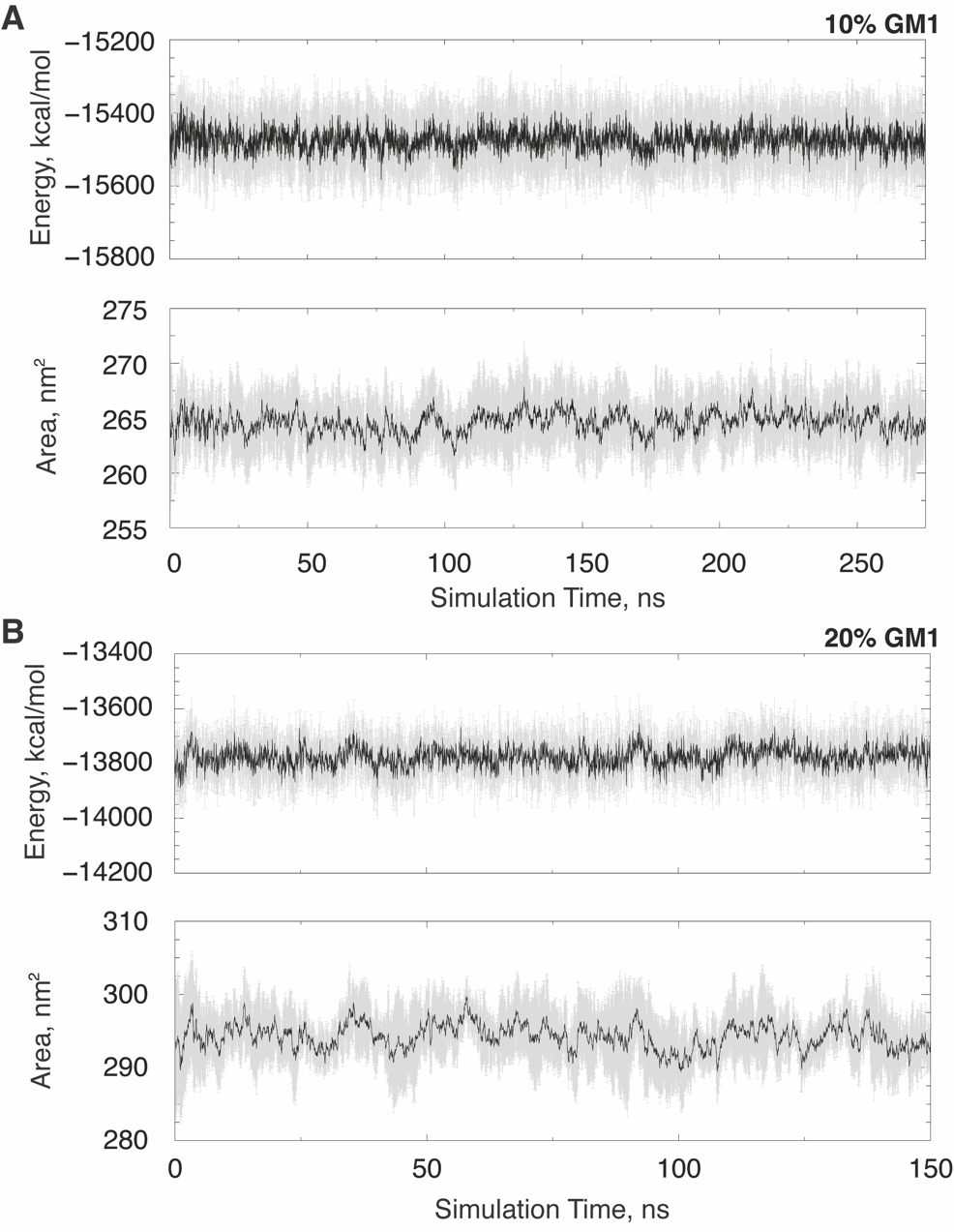


Figure S12. Equilibration evaluation of DMD simulations of POPC membranes mixed with (A) 10% and (B) 20% GM1. For each molecular system, the average value (black line) and standard deviation (gray error bars) for potential energy and membrane area are shown as a function of simulation time, based on ensemble averages over 10 independent simulations starting with randomized atomic velocities. The plots clearly indicate that our DMD simulations for both systems rapidly reached their respective steady states.
